# Incubation temperature shapes growth and mitochondrial metabolism across embryonic development in Japanese quail

**DOI:** 10.1098/rspb.2025.1752

**Published:** 2025-09-10

**Authors:** Elisa Thoral, Maria Gomez Correia, Imen Chamkha, Eskil Elmér, Andreas Nord

**Affiliations:** ^1^ La Rochelle Université - CNRS UMR 7266 LIttoral ENvironnement et Sociétés, 17000 La Rochelle, France; ^2^ Department of Biology, Evolutionary Ecology and Infection Biology, Lund University, SE-223 62, Lund, Sweden; ^3^ Department of Biological and Environmental Science, University of Jyväskylä, 40014 Jyväskylä, Finland; ^4^ Department of Clinical Sciences, Mitochondrial Medicine, Lund University, SE-221 84, Lund, Sweden; ^5^ Swedish Centre for Impacts of Climate Extremes (climes), Lund University, Lund, Sweden

**Keywords:** birds, citrate synthase, embryonic development, oxidative stress, reactive oxygen species, energy metabolism

## Abstract

Incubation temperature affects both growth and energy metabolism in birds after hatching. Changes in cellular mechanisms, including mitochondrial function, are a likely but unexplored explanation for these effects. To test whether temperature-dependent changes to mitochondria may link embryonic development to the post-natal phenotype, we incubated Japanese quail eggs at constant low (36.0°C), medium (37.5°C) or high (39.0°C) temperature and studied mitochondrial function and growth during embryogenesis and at hatching. Embryos grew faster and had higher mitochondrial metabolism at the high incubation temperature. Low incubation temperature slowed embryonic development and decreased phosphorylating respiration but was associated with higher adenosine triphosphate production efficiency. These respiration changes were mirrored by differences in mitochondrial content, which was the lowest in cold embryos. Neither treatment affected reactive oxygen species production. Hence, improved coupling efficiency in cold embryos may have partially compensated for lower adenosine triphosphate production without increasing oxidative stress. Size differences had disappeared by hatching. However, cold-incubated chicks had a higher mitochondrial content compared with the other groups. Our study suggests that thermal suppression of embryonic metabolism may be compensated by a combination of increased coupling, longer developmental time and late-occurring upregulation of mitochondrial content. The long-term implications of these results should be studied further.

## 1. Introduction

Avian incubation is subject to a trade-off between parental energy investment in egg heating and the thermal environment provided for developing offspring [1,2]. Accordingly, to maintain suitable developmental conditions in the nest [3,4], parents increase the metabolic rate when incubating larger clutches or in cold temperatures but engage in egg cooling in the warmth [5]. However, compensation is typically incomplete, causing egg temperature to drop or rise depending on the nature of the challenge to the nest thermal environment [3,4,6]. Even if bird embryos can buffer alterations to their thermal environment to some extent [7], prolonged variation in incubation conditions will invariably lead to changes in embryonic body and developmental temperature [8]. This can also have long-lasting effects on offspring phenotype [9,10].

Low incubation temperature reduces the rate of embryonic development [11], increases hatching failure [12] and is associated with smaller body mass and size after hatching [10,12]. By analogy, incubation at high temperature within the natural range of variation increases developmental rate and hatching success [13–15] and typically improves both residual yolk reserves at hatching [13–15] and subsequent body condition of chicks [16]. Hence, previous research suggests that embryonic development at low temperature reduces the effectiveness of tissue synthesis and increases the cost of development [9,17]. Variation in embryonic temperature also affects post-hatching physiology, most notably through upregulated metabolic rate in chicks that were incubated in hypothermic conditions [12,17], and improved body temperature control upon heat stress in chicks incubated at higher-than-normal temperature [18]. The physiological pathways linking embryonic energy turnover rate, incubation temperature and post-natal phenotype remain understudied. This is problematic, because lack of insight into underlying regulatory mechanisms risks leaving predictions about developmental consequences of environmental perturbations, including climate warming (cf. [19]), blunt or even erroneous.

Previous studies have investigated how thermal conditions during embryonic development affect the fundamental aspects of energy production at the cellular level, which could provide further insight into the mechanisms underlying incubation-temperature-dependent metabolic phenotypes. Indeed, more than 90% of cellular energy demand is covered by the production of adenosine triphosphate (ATP) through oxidative phosphorylation in the mitochondria [20]. Mitochondrial function can be epigenetically programmed by environmental perturbations during development (reviewed by Gyllenhammer *et al.* [21] and Koch *et al.* [22]). In line with this, studies on quail and chicken have demonstrated that exposure to elevated incubation temperatures during the second and third trimesters of embryonic development leads to diminished expression of several genes associated with mitochondrial function and the electron transport chain [23,24]. Moreover, incubation at high temperature increased both phosphorylating respiration and maximum uncoupled respiration capacity in the red blood cells of Japanese quail several weeks after hatching, with some effects of the embryonic environment remaining into adulthood [25]. While it is established that incubation temperature can influence mitochondrial respiration and function after hatching, less attention has been given to the early ontogeny of these effects. However, Krischek *et al.* [26] reported that elevated or reduced incubation temperatures during the second trimester in broilers, respectively enhanced or suppressed both phosphorylating respiration and aerobic enzyme activity in embryonic skeletal muscle. Nonetheless, the study did not examine whether these differences persisted at hatching or investigate the underlying mechanisms driving variation in mitochondrial respiration. Moreover, most previous studies have focused on agricultural settings, often with an implicit or explicit emphasis on production outcomes. As a result, our understanding of how avian embryonic mitochondria respond to ecologically relevant temperature variation, such as consistently elevated or reduced incubation temperatures throughout development reflecting parental energy investment (reviewed by Nord & Williams [1] and by Hepp *et al.* [27]), remains limited.

The coupling between ATP production and oxygen consumption is usually not complete, since oxygen is partly used to counteract proton leak across the inner mitochondrial membrane [28]. Theoretically, improved coupling could therefore compensate for the negative effect of low incubation temperature on embryonic development [29,30] by making ATP production more efficient for each oxygen molecule that is reduced during electron transport. However, reduced leak respiration potentially increases the fraction of electrons diverted to the production of reactive oxygen species (ROS) [22]. If ROS are not scavenged by antioxidant defences and are present in large quantities in the organism, they may cause oxidative damage to cells [31,32], which increases the rate of senescence [33]. An association between incubation temperature and coupling efficiency could explain why hypothermic incubation leads to both accumulation of oxidative stress over time [34] and a reduction in lifespan [35,36] (but see [37] for an exception). To the best of our knowledge, no previous study has addressed the hypothesis that mitochondrial coupling increases in cold-incubated embryos and that this could be associated with a higher oxidative stress.

Incubation temperature also affects the *response* to thermal stressors later in life. Studies on poultry show that chicks that are heat-challenged as embryos are more tolerant to heat stress after hatching and into adolescence [38–41], whereas chicks subjected to precisely timed, short, cold stimuli improve cold tolerance [42]. Work on non-poultry species reveals similarly strong effects on chick thermoregulatory responses, though the direction of change is typically the opposite [11,16,43]. However, it is not known how these organismal responses present at the cellular level. Research on ectotherms shows that thermal conditions may affect the mitochondrial response to acute changes in temperature [44,45]. For example, acclimation to high temperature reduces the thermal sensitivity of fish mitochondria, with an attenuated respiration increase in response to acute warming [46–48]. By analogy, studies of birds show that cold acclimation increases the thermal sensitivity of mitochondrial function in response to acute heat stress [49]. Measurement of how the thermal sensitivity of embryonic mitochondria is affected by incubation temperature might therefore elucidate the mechanistic link between thermal conditions experienced during development and organismal thermoregulation after hatching.

The aim of this study was to assess whether changes in mitochondrial function during embryonic development can provide a functional link explaining phenotypic effects of variation in incubation temperature before and after hatching. Here, we incubated eggs of Japanese quail (*Coturnix japonica*) across a biologically relevant range of low to high temperatures. We then measured embryonic growth and mitochondrial function in key metabolic tissues, as well as the thermal sensitivity of mitochondrial respiration and ROS production in response to acute warming. Finally, we investigated how incubation temperature affected the development of mitochondrial content between early embryogenesis and hatching. We expected that incubation at low temperature would cause a decreased mitochondrial respiration and a resultant reduction of growth rate. This could, however, be compensated by an increase in mitochondrial coupling efficiency, at the expense of increased ROS production. We also predicted that incubation at low temperature would render embryos more temperature-sensitive, reflected in more pronounced changes of mitochondrial respiration and ROS production in response to *in vitro* warming compared with embryos that were incubated at higher temperature.

## 2. Material and methods

**Figure 1.**
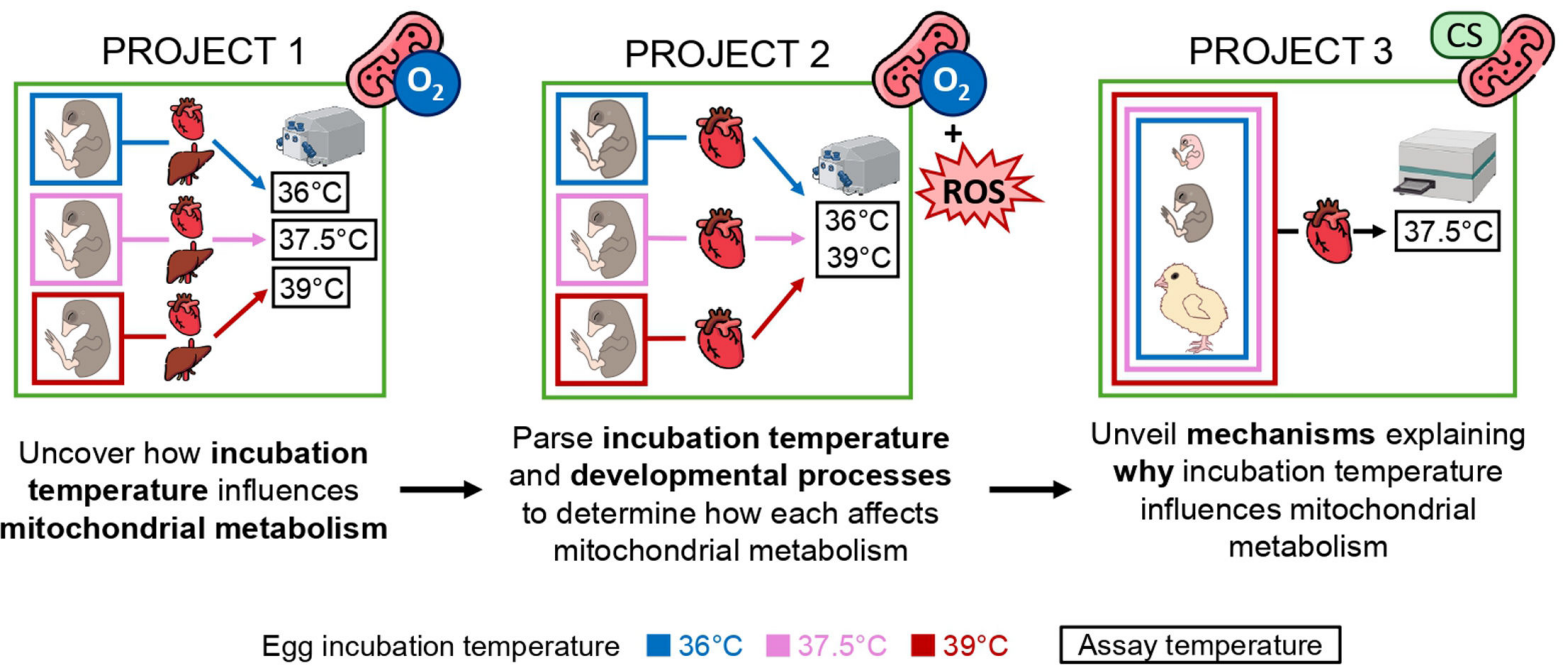
Schematic of the experiment. Projects 1 and 2 were performed when eggs had been incubated for 13 days, while Project 3 was performed after 7 and 13 days of incubation and at hatching. Measurements were performed in liver and/or heart tissue, as indicated in the panels. Biometric data were collected at each time point. ROS, reactive oxygen species; CS, citrate synthase.

We performed three complementary projects to uncover how constant thermal conditions during embryonic development affected growth and mitochondrial metabolism (figure 1). Firstly, we studied mitochondrial metabolism during representative *in ovo* conditions by measuring respiration in heart and liver tissue at incubation temperature (Project 1). Then, we studied if any variation in respiration traits was due to incubation temperature as such, or if it reflected variation in incubation-temperature-dependent developmental trajectories (Project 2). Finally, we asked when and how any differences between incubation temperature treatments arose, investigating the ontogeny of mitochondrial content in heart tissue (Project 3). The protocols of each project are detailed below.

### (a) Egg incubation

Japanese quail eggs were obtained from a commercial breeder (Helsingborgs Gårdsbutik, Allerum, Skåne, Sweden) and were subsequently used in three complementary projects to assess temperature-dependent mitochondrial metabolism (Projects 1 and 2) and embryonic growth and aerobic enzymatic activity (Project 3) in different tissues (see electronic supplementary material, table S1, for details). Immediately upon delivery to Lund University, the eggs were placed on an egg turner at 19°C for 1−7 days. Eggs were then randomly distributed across three incubators (Brinsea Ova-Easy 380 Advance EX) set at 36.0°C (‘cold’ treatment), 37.5°C (‘medium’ treatment) or 39.0°C (‘warm’ treatment), and 50% relative humidity (maintained using the Ova-Easy/TLC Advance Humidity Pump, Brinsea). We decided to collect embryos at similar chronological ages in all treatments, instead of similar development stages (except for hatchlings), assuming that the effect associated with thermal treatments on metabolism would be greater and would compensate for the effect associated with a difference in developmental stages at a similar age. The differences in degree days for all chronological ages (except hatchlings) are presented for each thermal treatment and project in electronic supplementary material, table S1. One incubator was set at one temperature for all three projects. Temperature was monitored daily using iButton data loggers (model DS1922L; accuracy ±0.5°C; Analogue Devices, Wilmington, MA) and was adjusted as needed to remain at target (electronic supplementary material, table S1). Fertilization was monitored by candling on day 7 of incubation, and infertile eggs were removed from the incubators. A total of 657 eggs were incubated, of which 430 were fertile. Of these, 254 were used for the experiment, and 176 were frozen for subsequent use in a different study (electronic supplementary material, table S1). Sixty-two eggs were used to study developmental temperature-dependent metabolism in heart and liver tissue (Project 1), 102 were used to measure the thermal sensitivity of mitochondrial respiration and ROS production in heart tissue (Project 2), and 90 were used to measure age- and temperature-dependent embryonic growth and citrate synthase (CS) activity in the heart (Project 3).

### (b) Tissue collection and measurement of embryonic growth rate

Tissues were harvested on day 7 (Project 3), day 13 (Projects 1−3) or at hatching (Project 3). The eggs were removed from the incubators and were submerged fully in ice for a minimum of 20 min to anaesthetize the embryos. The eggs were then opened with scissors and both the embryo and yolk were quickly weighed with a precision scale (KERN ABS-80-4N; accuracy ±0.1 mg) within 30 s of opening the egg, after which the embryos were decapitated. Hatchlings (Project 3) were removed from the incubator between 1 and 14 h after hatching. They were weighed when completely dry and then euthanized using inert gas (nitrogen; [50]).

Embryos and hatchlings were dissected in a Petri dish on ice under a binocular microscope (×60 magnification). The entire heart (Projects 1−3) and a liver sample (Projects 1 and 3) were collected and weighed, immediately placed in ice-cold respiration medium (MiR05: 0.5 mM EGTA, 3 mM MgCl_2_, 60 mM K-lactobionate, 20 mM taurine, 10 mM KH_2_PO_4_, 20 mM HEPES, 110 mM sucrose, 1 g l^−1^ free-fatty-acid bovine serum albumin, pH 7.1) and were then stored on ice until further processed. In Project 3, the entire liver and heart were weighed, put on ice and preserved at −20°C within 10 min. These samples were transferred to −80°C within 6 h, where they were stored until processed. We also excised and weighed the internalized yolk sac in Project 3 hatchlings.

### (c) Measurement of mitochondrial respiration and H_2_O_2_ release

Mitochondrial metabolism was assessed using Oxygraph O2k high-resolution respirometers (Oroboros Instruments, Innsbruck, Austria). The respirometers were calibrated every morning at air saturation oxygen levels and at the desired temperature (36.0, 37.5 or 39.0°C). The instrument/temperature combination was changed between days. Data were acquired using DatLab v. 7 (Oroboros Instruments).

In Project 1, we assessed mitochondrial respiration at incubation temperature. Embryos were collected at day 13 of incubation. Mitochondrial respiration was measured in both liver and heart tissue. Both tissues were homogenized using microdissection scissors in MiR05 until a homogeneus solution had been obtained following [51].

The homogenates were diluted in MiR05 to a final concentration of 1 mg ml^−1^ for the liver and 0.5 mg ml^−1^ for the heart, which provided optimal signal-to-noise ratios (determined in pilot studies). Then, 2.1 ml of the diluted solution was placed directly in the respirometry chambers, and a substrate-uncoupler-inhibitor titration (SUIT) protocol was used to address mitochondrial function (electronic supplementary material, table S3). Briefly, glutamate (chamber concentration: 5 mM) and malate (5 mM) were injected to activate complex I (i.e. L(n) respiration). Adenosine diphosphate (ADP; 1 mM) was then injected to activate phosphorylating (i.e. ATP-producing) respiration through complex I (i.e. OXPHOS_CI_ respiration). Then, we injected succinate (10 mM) to activate complex II, after which we measured the maximum phosphorylating capacity of the electron transport system (i.e. OXPHOS_CI+CII_ respiration). Oligomycin (1 µg ml^–1^) was then used to inhibit ATP synthase and measure proton leak respiration (i.e. L(Omy)). The uncoupled respiration (i.e. electron transport system (ETS) respiration) was then obtained by titrating carbonyl cyanide-*p*-trifluoro-methoxyphenyl hydrazine until maximum oxygen consumption (0.25−0.5 µl per injection). Finally, antimycin A (1 µg ml^−1^) was injected to inhibit complex III and measure non-mitochondrial respiration (ROX). The integrity of the outer mitochondrial membrane was assessed by the addition of cytochrome *c* (10 µM) after the measurement of OXPHOS_CI+CII_. The cytochrome *c* effect was low (mean ± s.d.: 6 ± 3%; range: 0–18%), suggesting high integrity of the mitochondrial membrane [52].

In Project 2, we tested whether incubation temperature affected the thermal sensitivity of mitochondrial respiration and ROS production (i.e. H_2_O_2_ release [53]). Data were collected on day 13 of incubation. We worked on heart tissue only, since liver constituents interfere with the measurement of ROS production [54]. The thermal sensitivity of mitochondrial metabolism was assessed by acutely exposing the tissue samples to low (i.e. 36°C) or high (i.e. 39°C) assay temperatures, representing the extreme ends of our incubation thermal treatments.

Heart samples were homogenized in ice-cold MiR05 using a Potter-Elvehjem grinder to allow injection of the homogenate into the closed chambers of the oxygraphs. To study thermal sensitivity, each homogenate (0.5 mg ml^–1^ chamber concentration) was assayed at both 36 and 39°C (i.e. the lowest and highest incubation temperatures in our study). H_2_O_2_ release was indexed as fluorescence detection of the extrinsic fluorophore Amplex^®^ Ultrared (AmR) [55]. The reaction of H_2_O_2_ and AmR was catalysed by horseradish peroxidase (HPR), which produces the red fluorescent compound resorufin. The change of emitted fluorescence intensity is directly proportional to the concentration of H_2_O_2_ [55]. This, in turn, represents the balance between H_2_O_2_ production and degradation, the latter of which could be influenced by endogenous antioxidant enzymes [56]. We will refer to this measure as ‘H_2_O_2_ release’ throughout the rest of this manuscript.

Briefly, AmR (10 µM), HPR (5 U ml^–1^) and superoxide dismutase (SOD; 50 U ml^–1^) were added to the respirometry chambers. Then, the rate of ROS production was measured as the molar rate of H_2_O_2_ appearance, using the titration of exogenous H_2_O_2_ to calibrate the fluorescent resorufin signal (0.1 mM per injection). The heart homogenate was then injected into the chambers. Titration of exogenous H_2_O_2_ was then performed again to calibrate the signal after the addition of the tissue sample, after which we used the SUIT protocol above to assess mitochondrial respiration. Titration of exogenous H_2_O_2_ was performed again after the injection of antimycin A to calibrate the signal.

### (d) Citrate synthase activity

In Project 3, we tested the hypothesis that any temperature-related changes in embryonic growth or mitochondrial metabolism could be explained by mitochondrial content. This was achieved by measuring CS activity in heart tissue throughout development in each incubation temperature group. CS, a key enzyme of the citric acid cycle [57], is a commonly used marker of mitochondrial density [48,58,59]. We performed a preliminary study to determine the optimal conditions for assessing CS activity in heart tissue at different ages. Details are disclosed in electronic supplementary material, figures S2–S5.

CS activity was measured at 37.5°C in a subset of samples (*n* = 10 per incubation temperature treatment and age) using a microplate reader (BIO-RAD Model 680 Microplate Reader) and the CS Activity Assay Kit from Sigma (EC no. 232-821-7, Sigma-Aldrich, USA). CS activity was determined using a coupled enzyme reaction between acetyl coenzyme A (acetyl-CoA) and oxaloacetate (OAA), resulting in a colorimetric product proportional to the enzymatic activity of the sample. The samples were thawed on the day of the enzymatic activity measurements, a small piece was cut off, weighed (sample mass range at day 7: 1.3−8.3 mg; at day 13: 12.6−48.6 mg; at hatching: 37.2−137 mg) and transferred to 600 µl of ice-cold homogenizing buffer (100 mM KH_2_PO_4_ buffer, pH 7.2, containing 1 mM EGTA, 1 mM EDTA, 0.1% Triton X-100 and 1 mM phenylmethylsulfonyl fluoride). Samples were then shredded using a tissue shredder (Polytron 10-35 GT, Kinematica) at 10 000 r.p.m. for 2 × 5 s. The homogenate was then diluted to 0.1 mg ml^–1^ before being centrifuged at 10 000*g* for 5 min. CS activity was assayed using the supernatant. Briefly, 50 µl of sample was added in each well with 140 µl of the Regent Mix (1× assay buffer from the kit, 30 mM acetyl-CoA, 1 mM 5,5′-dithiobis(2-nitrobenzoic acid) (DTNB)). After measuring the background at 412 nm at 37°C for 2 min, 10 µl of OAA (10 mM) was injected into the wells and the CS activity was recorded over the following 20 min. The background value was removed from the CS activity if the slope was visually linear, which coincided with *R*^2^ > 0.7. The background value was set as null if the slope was considered nonlinear upon visual inspection (associated with 0 < *R*^2^ < 0.69). All samples were randomized between ages and incubation temperature treatments. All samples were run in triplicate, and we used mean CS activity in all analyses. The CS activity was expressed in µmol OAA × mg^–1^ of heart tissue × min^–1^.

### (e) Data analyses

For the mitochondrial respiration, ROX was removed from all the other respiration rates before analyses. We calculated the net phosphorylation efficiency (Net P) and the fractional electron leak (FEL; see electronic supplementary material, table S3).

In Project 2, four individuals were removed from the statistical analyses of mitochondrial respiration owing to erroneous calculation of sample concentration.

All statistical analyses were performed using R v. 4.2.3 [60]. Firstly, we tested the effect of age (day 7, day 13 or hatching) and incubation temperature (cold, medium or warm) on morphological parameters (body mass, yolk mass, liver mass, heart mass and yolk : body mass ratio) using generalized linear models with a gamma distribution and log link (glm() function in the *stats* package). Incubation temperature, age and their interaction were used as factors. These models were run using data from Project 3. Growth characteristics of embryos from Projects 1 and 2 are also presented in electronic supplementary material, table S2.

Secondly, we tested how mitochondrial respiration traits (L(n), OXPHOS_CI_, OXPHOS_CI+CII_, L(Omy), ETS and Net P) in liver and heart differed between treatments when assayed at their respective incubation temperatures (i.e. Project 1). This was achieved using linear models (lm from the *stats* package), using each mitochondrial parameter as a dependent variable, and treatment as an explanatory variable. Thirdly, we used linear mixed models (lme() from the *lme4* package [61]) to model the thermal sensitivity of mitochondrial respiratory capacity and H_2_O_2_ release (at 36 and 39°C, i.e. Project 2). Mitochondrial traits (respiration rates and H_2_O_2_ release parameters) were included as dependent variables, while incubation temperature (cold, medium and warm), assay temperature (36 or 39°C) and their interaction were included as explanatory variables. The residuals in the OXPHOS_CI_, OXPHOS_CI+CII_, H_2_O_2_ release and FEL models were not normally distributed. Hence, these data were analysed using a generalized linear mixed effects model with a gamma distribution and a log link (glmer() function in the *lme4* package). All other models from this dataset were fitted with a Gaussian distribution. CS activity was analysed using linear models (lm from *stats* package), using CS as the dependent variable, and incubation temperature, age and incubation treatment × age as explanatory variables. Body mass (mean-centred by incubation temperature) was used as a covariate in all models describing mitochondrial respiration and CS activity since it may be related to mitochondrial metabolism [62,63].

All parameters were retained in the models regardless of their significances. Estimates of the fixed effects were calculated using the emmeans() function from the *emmeans* package [64]. When the interaction was significant, we performed post hoc tests using the emmeans() function from the *emmeans* package. When the interaction was significant, *post hoc* tests were performed between incubation temperature treatments within ages or between assay temperature within incubation temperature treatments.

## 3. Results

### (a) Effects of incubation temperature on embryonic growth and incubation period

**Figure 2.**
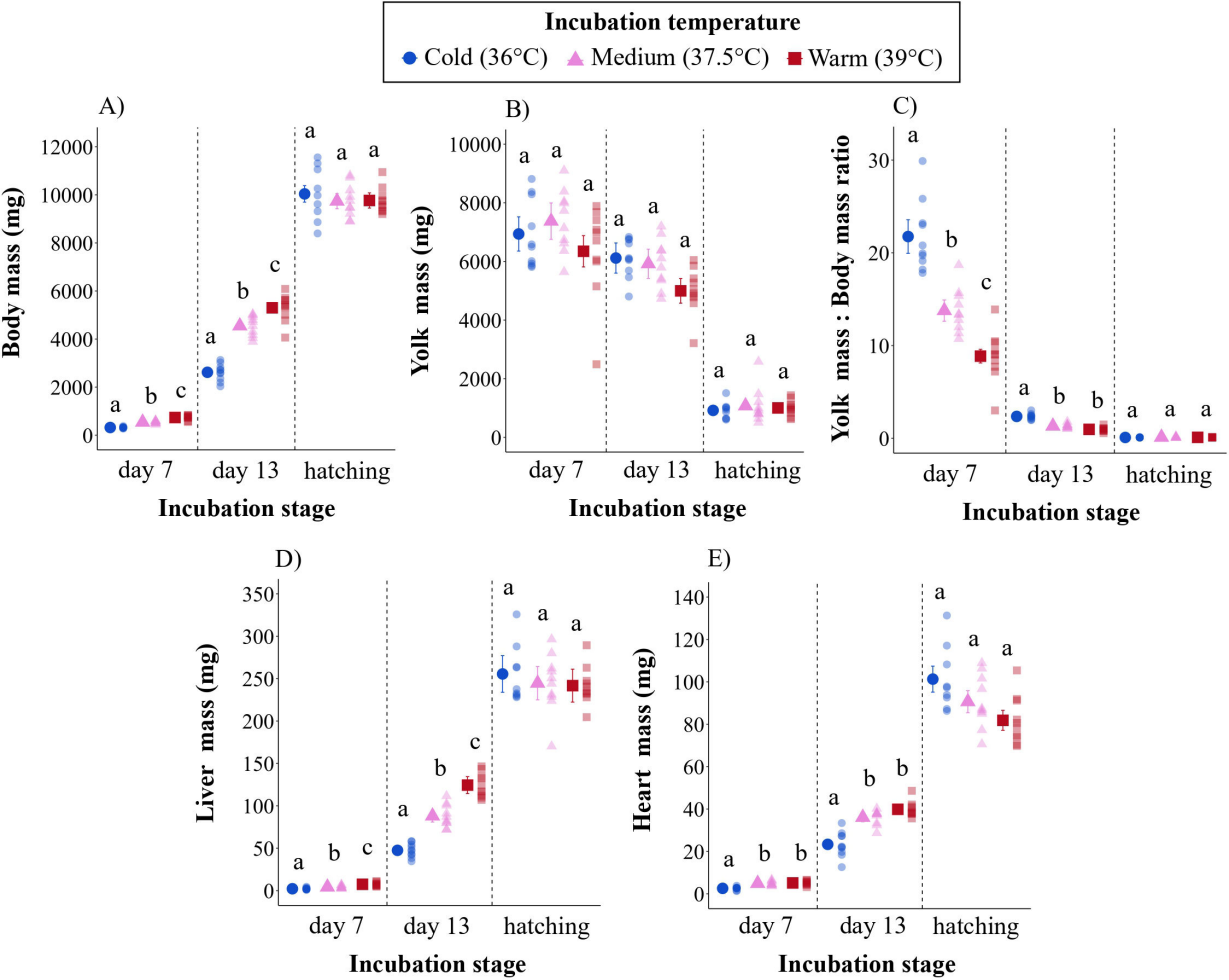
Morphological parameters at days 7 and 13 of embryonic development and at hatching for Japanese quail embryos incubated at cold, medium or warm temperature. The solid plotting symbols are estimated means ± s.e.m. Paler points are raw data. Different lowercase letters show significant differences between treatments within ages. All parameters were significantly different between ages across treatments.

Incubation temperature significantly affected the development of all morphological traits in a nonlinear, treatment-specific manner (figure 2).

Body mass, liver mass, heart mass and the yolk : body mass ratio were all affected by incubation temperature, but the effect differed between ages (incubation temperature × age: all *p* < 0.001). Specifically, after 7 days of incubation, body mass and liver mass were 56 and 70% higher, respectively, in embryos incubated at warm temperature compared with embryos incubated at cold temperature, and 27 and 44% higher, respectively, in warm compared with medium embryos. Heart mass was also higher, by 50%, in warm embryos compared with cold embryos, and by 47% in medium embryos compared with cold embryos. After 13 days of incubation, body mass and liver mass were also 51 and 62% higher in warm compared with cold embryos, and 14 and 29% higher in warm compared with medium embryos. An increase in heart mass of 41 and 35% was also observed in warm and medium embryos, respectively, on day 13 compared with cold embryos. Liver and body mass never differed significantly between cold and medium embryos, and heart mass never differed between medium and warm embryos.

Yolk mass was significantly affected by age, decreasing progressively during embryonic development, independently of the incubation temperature treatment (figure 2B). However, after 7 and 13 days of incubation, the yolk : body mass ratio was significantly lower in the warm group compared with the cold and medium groups (electronic supplementary material, table S4; figure 2C). After 13 days, the proportion of yolk was still lower in medium and warm treatments compared with cold treatment.

At hatching, none of the temperature-dependent morphological differences present during embryonic development remained (all pairwise *p* > 0.700; electronic supplementary material, table S4; figure 2).

The incubation period increased with decreasing incubation temperature, being 1.7 days longer in the medium (18.3 ± 0.2 days) compared with the warm (16.6 ± 0.2 days) temperature, and an additional 2.6 days when incubated at a cold temperature (20.9 ± 0.2 days).

### (b) Effects of incubation temperature on mitochondrial respiration

**Figure 3.**
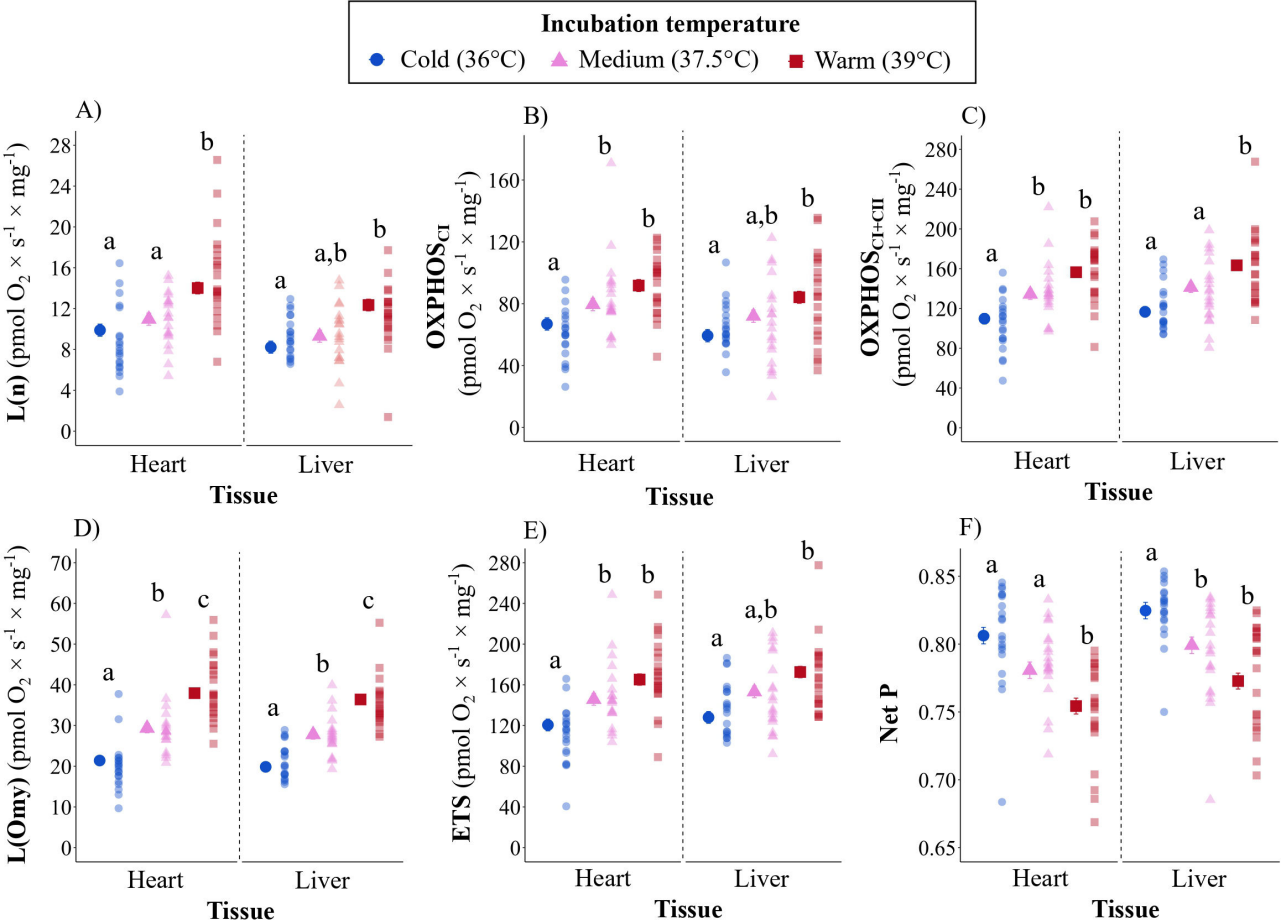
Mitochondrial respiration traits measured at incubation temperature (cold: 36.0°C; medium: 37.5°C; warm: 39.0°C) in heart or liver tissue of Japanese quail at day 13 of incubation for (A) L(n) respiration, (B) OXPHOS_CI_ respiration, (C) OXPHOS_CI+CII_ respiration, (D) L(Omy) respiration, (E) ETS respiration, (F) net phosphorylation efficiency (Net P, i.e. the mitochondrial efficiency to produce ATP). The solid plotting symbols are estimated means ± s.e.m. Paler points are raw data. Different lowercase letters show significant differences between assay temperatures.

**Figure 4.**
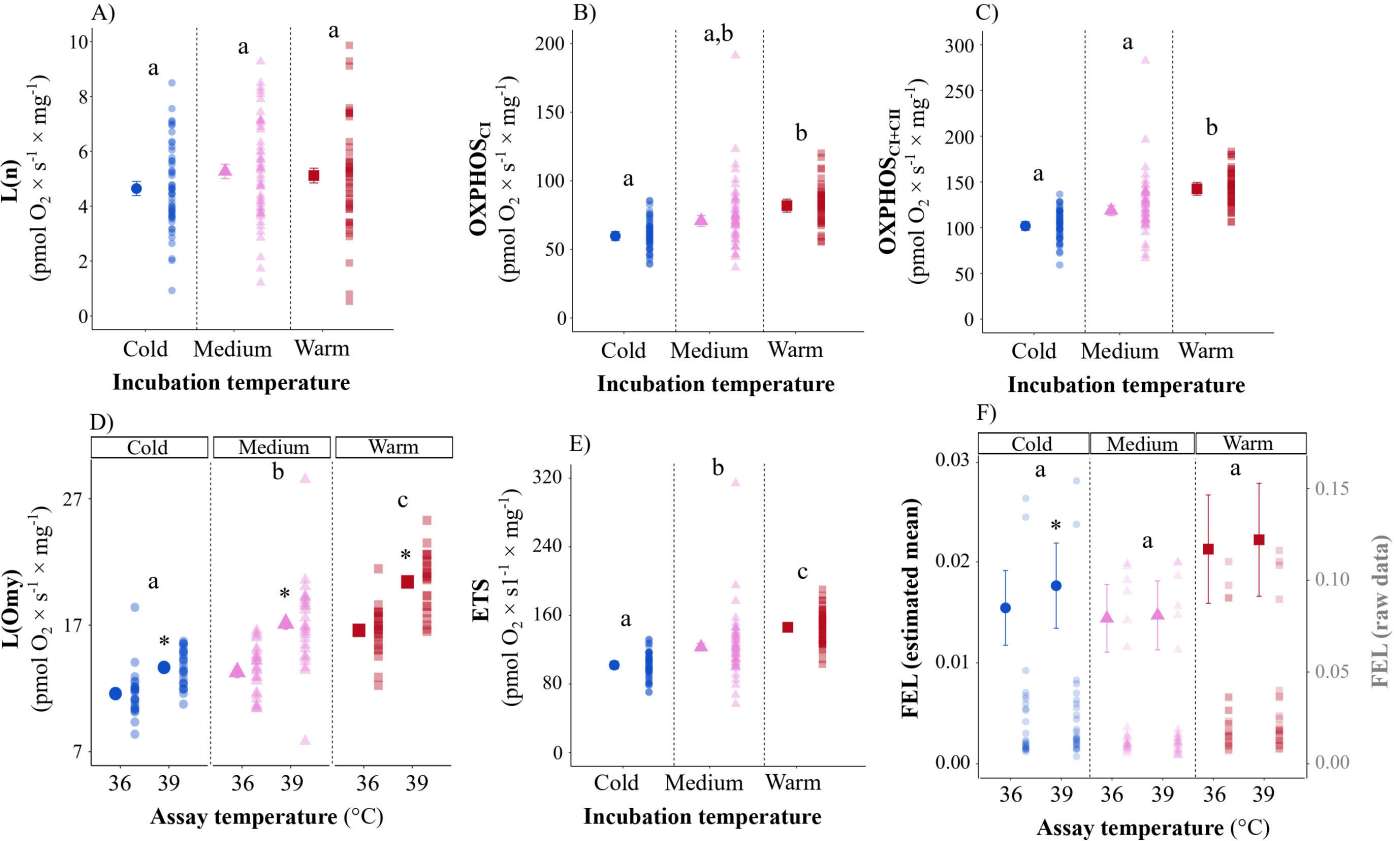
Mitochondrial metabolism parameters (respiration and reactive oxygen species production) in heart tissue from Japanese quail embryos incubated at cold (36°C), medium (37.5°C) or warm (39.0°C) temperature at day 13 of incubation. Measurements were made at 36 and 39°C for all samples. Solid plotting symbols show estimated means ± s.e.m. When the interaction between treatment and assay temperature was not significant, we plotted the estimated main effects of treatment (i.e. incubation temperature) and denoted significant group differences by different lowercase letters. When the interaction was significant (D,F), we calculated treatment estimates for each assay temperature, with different lowercase letters showing differences between treatments, and an asterisk showing the differences between assay temperatures within treatments. Paler points are raw data. For (F), the left *y*-axis corresponds to estimated means (solid plotting symbols), while the right *y*-axis corresponds to raw data (semi-transparent points). (A) L(n) respiration, (B) OXPHOS_CI_ respiration, (C) OXPHOS_CI+CII_ respiration, (D) L(Omy) respiration, (E) ETS respiration, (F) fractional electron leak (FEL) in the L(Omy) state.

There was a clear incubation temperature dependence of all mitochondrial respiration parameters in both heart and liver tissue after 13 days of incubation (figures 3 and 4; electronic supplementary material, tables S5 and S6).

In the heart, L(n) respiration was 41 and 28% higher in embryos incubated at warm temperatures compared with cold and medium embryos, respectively, but there was no difference between cold and medium embryos (figure 3A). In the liver, L(n) respiration was 18% higher in embryos incubated at warm temperature compared with cold temperature (figure 3A). In both tissues, L(Omy) increased significantly with each incubation temperature group (figure 3D).

OXPHOS_CI_, OXPHOS_CI+CII_ and ETS respiration were 35, 34 and 35% lower in heart tissue from embryos incubated at cold temperature compared with warm embryos. These parameters were also 26 (OXPHOS_CI_), 25 (OXPHOS_CI+CII_) and 26% (ETS) lower in cold embryos compared with medium embryos (figure 3B,C,E). However, there was no significant difference in any of these traits between medium and warm embryos (0.068 ≤ *p* ≤ 0.345). In liver tissue, OXPHOS_CI_, OXPHOS_CI+CII_ and ETS were also significantly lower in cold embryos compared with warm embryos (by 18–23%; figure 3B,C,E), but we observed no significant difference between cold and medium embryos (see electronic supplementary material, table S6). Moreover, OXPHOS_CI+CII_ was significantly higher in warm embryos compared with medium embryos, but there was no difference between these treatments in OXPHOS_CI_ (*p* = 0.084) and ETS (*p* = 0.165; see figure 3B,C,E).

Finally, Net P (i.e. the relative coupling of electron transport to ATP production) was significantly lower in the heart of warm embryos, where 75% (i.e. net *p* = 0.75) of the oxygen consumed was related to phosphorylation, compared with 81% in the cold and 79% in the medium embryos (which did not differ; figure 3F). In the liver, Net P was significantly lower in medium (79%) and warm embryos (78%) compared with cold embryos (84%; figure 3F).

### (c) Thermal sensitivity of mitochondrial respiration and H_2_O_2_ release

Across assay temperatures, OXPHOS_CI_, OXPHOS_CI+CII_ and ETS were affected by the incubation treatment, being 27–30% higher in warm compared with cold embryos, and 13–15% higher compared with medium embryos (figure 4B,C,E). Medium embryos, in turn, had 18% higher ETS compared with cold embryos, and a similar tendency was observed for OXPHOS_CI+CII_ (*p* = 0.054). The effect of incubation temperature on the thermal sensitivity of mitochondrial respiration was only evident for L(Omy), being higher in medium and warm embryos (27 and 23% increase, respectively, at 39°C) compared with lower in cold embryos (17% increase at 39°C) (figure 4D).

H_2_O_2_ release measured during the L(Omy) state were higher at 39°C compared with 36°C (by 35%), but it was not affected by incubation temperature (electronic supplementary material, table S7 and figure S1) nor by the interacting effect of incubation and assay temperatures (*p* = 0.18). However, the thermal sensitivity of FEL, i.e. ROS production relative to the total mitochondrial electron flux, was *higher* in cold embryos compared with the two other treatments, increasing by 15% when the assay temperature increased from 36 to 39°C, compared with 4% in the warm and medium embryos (figure 4F).

### (d) Effect of incubation temperature on citrate synthase activity

**Figure 5.**
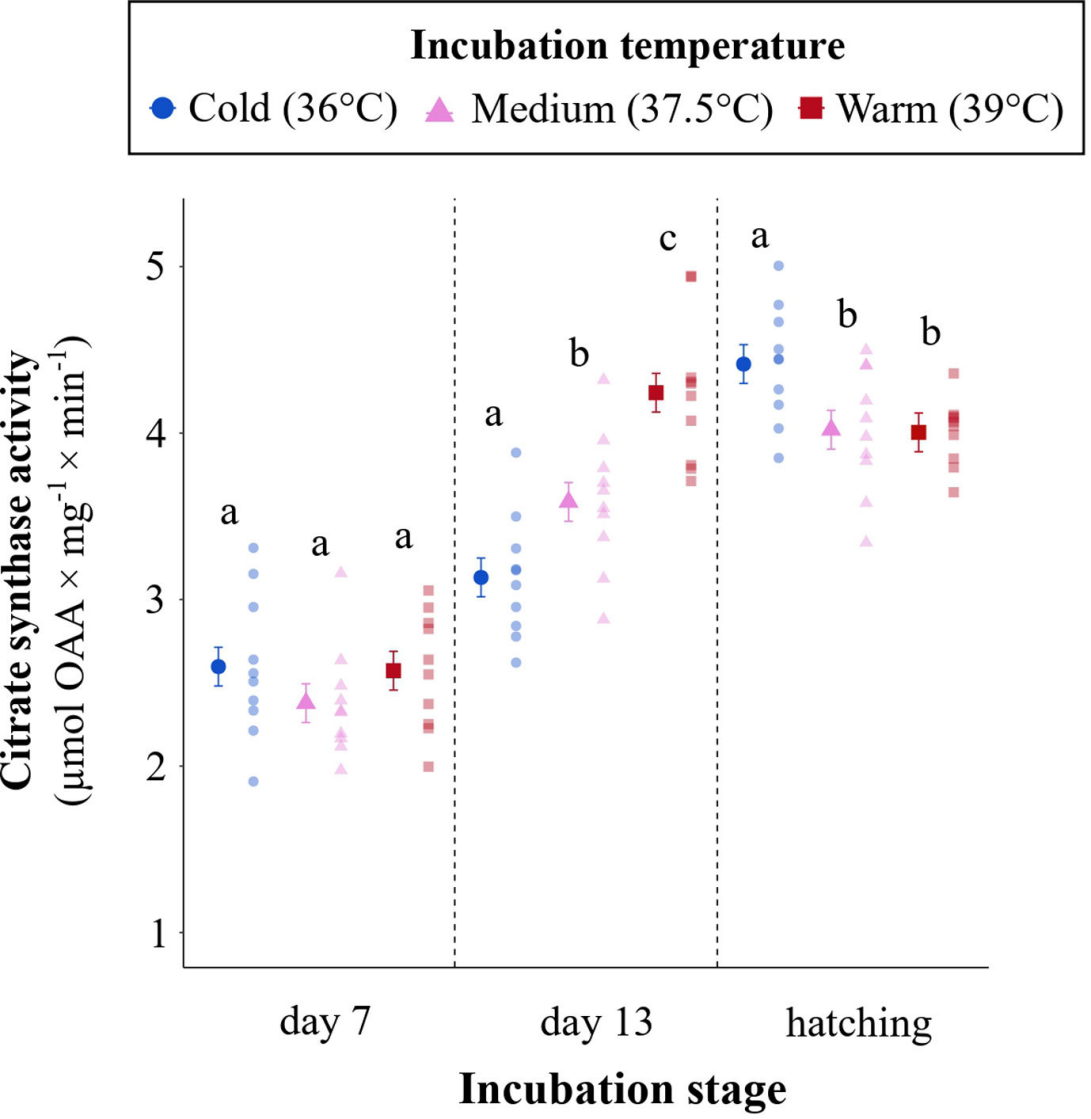
Ontogeny of mitochondrial content (approximated by citrate synthase (CS) activity) as a function of incubation temperature (cold: 36.0°C; medium: 37.5°C; warm: 39.0°C). The solid plotting symbols are estimated means ± s.e.m. Paler points are raw data. Different lowercase letters show significant differences between incubation temperatures within ages.

CS activity increased with age in an incubation-temperature-specific manner (incubation temperature × age: *p* < 0.001; figure 5; electronic supplementary material, table S8). There was no difference in CS between groups on day 7. However, by day 13, CS activity in the warm group was 26 and 15% higher, compared with the cold and medium groups, respectively. Moreover, CS activity was 13% higher in medium compared with cold embryos. Conversely, at hatching, CS activity was *higher* in cold embryos compared with medium and warm embryos (*p* = 0.049 and 0.039, respectively) (figure 5).

## 4. Discussion

Thermal conditions experienced during embryonic development caused changes in growth and energy metabolism in quail embryos. Specifically, a reduction in incubation temperature slowed embryonic development but increased coupling efficiency in both liver and heart and decreased thermal sensitivity of L(Omy) respiration relative to the higher incubation temperatures. Overall, mitochondrial respiration of embryos was higher in the higher incubation temperature treatments, likely as a consequence of a higher mitochondrial content in these groups. At hatching, there were no remaining differences in morphological parameters or cellular respiration traits, but cold-incubated chicks maintained higher mitochondrial content compared with the other treatment groups.

### (a) Lower cellular metabolism hinders growth rate in cold-incubated embryos

In line with previous studies [11,34,65], low incubation temperature increased the incubation period (approx. 20.9 days) compared with medium (approx. 18.3 days) and warm temperatures (approx. 16.6 days). This was presumably a consequence of a temperature-induced reduction of growth rate, which was observed at both organismal and organ levels during the first two trimesters of embryonic development (figure 2). Cold embryos presented a higher yolk : body mass ratio, indicating a lower utilization of the yolk reserves during incubation, corroborating a lower energy conversion efficiency in this group. Our results show that a reduction in mitochondrial respiration and associated ATP production is a possible mechanism explaining why low incubation temperature reduced embryonic development rate, both when measured at incubation temperature (i.e. in Project 1) and when measured under identical thermal conditions (i.e. in Project 2) after 13 days of embryonic development. Indeed, at a same chronological age, cold embryos presented lower mitochondrial metabolism. Even if the thermal load differed between thermal treatments experienced during incubation (see electronic supplementary material, table S1), the effect size widely surpasses expectations based on the number of degree days alone. Studies on the chicken have found similar effects of low incubation temperature on mitochondrial respiration in embryonic skeletal muscle [26]. Accelerated development of mitochondrial content (here inferred from CS activity) is the most parsimonious explanation for differences in respiration between the treatments: by day 13 of incubation, the warm and medium embryos had largely reached the mitochondrial content of hatchlings, whereas the most pronounced increase in CS activity in cold embryos occurred during the third trimester (figure 5).

While cold embryo mitochondria were slower overall, our study suggests that acclimation to low temperature causes metabolic changes that enhance mitochondrial function. Firstly, mitochondria from cold embryos were more tightly coupled compared with the medium and warm embryos (i.e. Net P was higher; figure 3F). It is therefore likely that cold embryos produced more ATP for a given respiration rate, partly compensating for thermal suppression of metabolism. Comparable results have been obtained in the context of growth rate in fish [66] and in response to acute *in vitro* cooling in both birds and ectotherms [30,67]. Moreover, L(Omy) respiration, which mainly counteracts proton leakage, was less affected by *in vitro* warming in cold embryos compared with other incubation thermal treatments. L(Omy) is regulated by active and passive processes, such as uncoupling proteins [68,69], membrane composition [70] and potential [71], and membrane fluidity [72], all of which are known to change in response to changes in environmental temperature [73]. The observed changes to leak respiration could therefore counteract negative effects of low temperature, including metabolic suppression [74] and decrease enzymatic substrate affinity [75]. Similar reductions in the thermal sensitivity of L(Omy) in response to cold acclimation have previously been obtained in the heart of Atlantic killifish *Fundulus heteroclitus* [76]. However, the reverse was observed in the liver of the same fish species [77]. Thus, affirmative conclusions on changes to coupling as a compensatory mechanism for low temperature in ectothermic bird embryos are likely premature. This notion should be subject to scrutiny in a wider set of study species, as well as across a range of tissues with different *in vivo* functions.

Theory predicts that tighter coupling of the electron transport chain should be associated with an increase in mitochondrial ROS production (cf. [22]), which has been associated with accumulation of somatic damage across the animal kingdom. However, we found little effect of incubation temperature on mitochondrial ROS production. This suggests that the tighter coupling efficiency in the cold treatment did not carry any oxidative costs during embryonic development. Hence, accumulating oxidative damage in birds hatched from cold-incubated eggs, recorded in previous accounts [34], should either be of non-mitochondrial origin or emerge after hatching. Still, the thermal sensitivity of FEL was higher in cold-incubated embryos. That is, at least when facing increased assay temperature, more electrons were diverted to ROS production in the cold treatment. This could be related to the lower thermal sensitivity of L(Omy), because increased proton leak should reduce ROS generation [28]. The relationship between ROS generation, FEL, oxidative damage and antioxidant defences in bird embryos developing at different temperatures should be confirmed in future studies.

### (b) Similar body mass but different mitochondrial content at hatching

Although developmental rate was reduced at both cellular and organismal levels in embryos incubated at low temperature, body and internal organ mass were similar between groups at hatching. These results contrast with previous studies on birds, including Japanese quail, where size and body mass of juveniles often increase with increasing incubation temperature [11,14,16,78]. Other studies have shown no effect of incubation temperature on morphological parameters after hatching [34,79]. This has been suggested to reflect a threshold size below which embryos do not survive the pipping stage [79]. However, morphological differences may also emerge during juvenile development even if not present at hatching [12], perhaps reflective of post-natal energy metabolism. In view of this, it is clear that the impact of the duration and intensity of thermal changes during incubation on physiology and growth from hatching until maturity needs additional study.

While no morphological traits differed between treatments at hatching, CS activity in the heart was elevated in cold-incubated embryos (figure 5), suggesting that this group hatched with higher mitochondrial content. This corroborates previous research in the chicken, where CS activity and a marker of mitochondrial biogenesis (peroxisome proliferator-activated receptor gamma coactivator 1-alpha (PGC-1α)) was upregulated in liver tissue shortly before and after hatching [80]. Similar effects of low temperature on mitochondrial biogenesis occur in adult birds [81]. Our results could indicate that cold embryos boosted metabolism (e.g. by producing more ATP or amino acid intermediates), starting in the latter third of incubation, possibly as a compensatory response for low initial growth rate. Such an incubation-temperature-driven increase in aerobic capacity may explain why chicks hatching from cold-incubated eggs show elevated resting metabolic rate early in life [12,81,82]. At this stage, increased metabolic capacity could be beneficial for any compensatory growth and upon cold exposure, when low-temperature-incubated chicks initially risk inferior thermoregulatory control [11,43]. Thus, thermal conditions during embryonic development could lead to advantageous alterations in energy metabolism that compensate for a bad start in life. More studies are now needed on the short- and long-lasting effects of incubation temperature to disentangle compensatory from carry-over effects, including the links between aerobic respiration, ROS and lifespan.

## Data Availability

Data are available at [83]. Supplementary material is available online [84].
